# Induction of the Unfolded Protein Response during Bovine Alphaherpesvirus 1 Infection

**DOI:** 10.3390/v12090974

**Published:** 2020-09-02

**Authors:** Song Wang, Xiaomei Ma, Hongmei Wang, Hongbin He

**Affiliations:** 1 Ruminant Diseases Research Center, College of Life Sciences, Shandong Normal University, Jinan 250014, China; alexwsong@163.com (S.W.); ChitaMa666@163.com (X.M.); 2 Key Laboratory of Animal Resistant Biology of Shandong, College of Life Sciences, Shandong Normal University, Jinan 250014, China

**Keywords:** ER stress, UPR, BoHV-1, viral replication

## Abstract

Bovine herpesvirus 1 (BoHV-1) is an alphaherpesvirus that causes great economic losses in the cattle industry. Herpesvirus infection generally induces endoplasmic reticulum (ER) stress, and the unfolded protein response (UPR) in infected cells. However, it is not clear whether ER stress and UPR can be induced by BoHV-1 infection. Here, we found that ER stress induced by BoHV-1 infection could activate all three UPR sensors (the activating transcription factor 6 (ATF6), the inositol-requiring enzyme 1 (IRE1), and the protein kinase RNA-like ER kinase (PERK)) in MDBK cells. During BoHV-1 infection, the ATF6 pathway of UPR did not affect viral replication. However, both knockdown and specific chemical inhibition of PERK attenuated the BoHV-1 proliferation, and chemical inhibition of PERK significantly reduced the viral replication at the post-entry step of the BoHV-1 life cycle. Furthermore, knockdown of IRE1 inhibits BoHV-1 replication, indicating that the IRE1 pathway may promote viral replication. Further study revealed that BoHV-1 replication was enhanced by IRE1 RNase activity inhibition at the stage of virus post-entry in MDBK cells. Furthermore, IRE1 kinase activity inhibition and RNase activity enhancement decrease BoHV1 replication via affecting the virus post-entry step. Our study revealed that BoHV-1 infection activated all three UPR signaling pathways in MDBK cells, and BoHV-1-induced PERK and IRE1 pathways may promote viral replication. This study provides a new perspective for the interactions of BoHV-1 and UPR, which is helpful to further elucidate the mechanism of BoHV-1 pathogenesis.

## 1. Introduction

Bovine herpesvirus 1 (BoHV-1) is a member of the alphaherpesvirus subfamily [1,2], having a double-stranded DNA genome with a size of 135.3 kilobase pairs that encodes an estimated 73 open reading frames [1,3]. BoHV-1 is an important pathogen responsible for significant economic losses of the cattle industry worldwide [4,5,6,7,8,9,10]. Infection in cattle leads to a series of clinical syndromes, such as bovine respiratory disease, gastrointestinal symptoms, reproductive tract disease, conjunctivitis, abortions, and severe neonatal disease [11].

The endoplasmic reticulum (ER) is an important organelle for protein synthesis, folding, modification, and trafficking [12,13]. ER homeostasis is tightly regulated, and glucose starvation, calcium dysregulation or aggregation of misfolded proteins in the ER can disrupt ER function resulting in ER stress [14,15]. The cells activate the unfolded protein response (UPR) to restore ER homeostasis [16]. There are three key UPR signal sensors: the activating transcription factor 6 (ATF6), the inositol-requiring enzyme 1 (IRE1), and the protein kinase RNA-like ER kinase (PERK) [17]. When there is the accumulation of misfolded or unfolded protein in the ER lumen, glucose-regulated protein 78 (GRP78) is released from the three UPR sensors, which results in the activation of three sensors and initiation of the UPR signaling cascades. ATF6 egresses from the ER and translocates to the Golgi apparatus for maturation through protease cleavage to generate ATF6f, which regulates UPR target gene expression [16,18,19]. The UPR signal (IRE1) involves the splicing of the x-box binding protein 1 (XBP1), which cleaves the 26-bp intron XBP1 and increases expression of its downstream target genes [20]. The activated kinase activity of PERK leads to phosphorylation of translational initiation factor eIF2α, resulting in inhibition of mRNA translation. Phospho-eIF2α promotes the induction of activating transcription factor 4 (ATF4), which stimulates the transcription of DNA damage inducible transcript 3 (DDIT3) and apoptosis endues [21,22].

Viruses completely rely on the host’s cellular mechanisms to synthesize a large number of viral proteins that destroy the cellular translation machinery and can cause ER stress and the UPR [23,24,25,26]. Studies have shown that the interaction of dengue virus envelope protein with GRP78, calreticulin, and calnexin promote proper folding and assembly of viral proteins for replication [27]. The UPR induced by porcine reproductive and respiratory syndrome virus (PRRSV) suppresses viral replication and RNA synthesis [28]. PERK and IRE1, but not the ATF6 pathway, can be activated by HCMV lytic replication [29]. Alpha herpesvirus replication also triggers ER stress [30]. Herpes simplex virus-1 (HSV-1) infection activates the ER-resident kinase PERK, ATF6 cleavage, and the IRE1 pathway [31,32,33]. The glycoprotein of varicella-zoster virus (VZV) differentially activates the unfolded protein response in infected cells [34]. However, induction of the UPR activation by BoHV-1, or the function played by UPR during BoHV-1 replication, remain unclear.

Published studies have reported that GRP78, a marker protein of ER stress [35], is up-regulated in Madin–Darby bovine kidney (MDBK) cells at the early stage of BoHV-1 infection using proteomic and phosphoproteomic tools [36]. However, the induction of the UPR has also not been researched during the BoHV-1 infection. We speculated that BoHV-1 infection induced ER stress to activate UPR. Consequently, we determined which branches of the UPR were activated during BoHV-1 infection, and investigated the role of the three UPR arms in regulating viral replication. In this study, we reported for the first time that BoHV-1 induces ER stress and activates all three UPR branches. We further uncovered that the ATF6 arm of the UPR does not affect BoHV-1 replication in MDBK cells and the PERK branch of the UPR promotes viral replication at the post-entry step of the BoHV-1 life cycle. Furthermore, we used small interfering RNAs and chemicals to inhibit IRE1 and found BoHV-1 replication was improved, which was related to IRE1 RNase activity inhibition. These findings improve our understanding of the BoHV-1–UPR interaction.

## 2. Materials and Methods

### 2.1. Cell Lines and Virus

Madin–Darby Bovine Kidney (MDBK) cells were obtained from ATCC CCL-22 and cultured in Dulbecco’s modified Eagle’s medium (DMEM, Gibco, Waltham, MA, USA) supplemented with 10% fetal bovine serum (FBS, Gibco, Waltham, MA, USA) and antibiotics (100 μg/mL streptomycin and 100 U/mL penicillin), and maintained in a humidified environment (37 °C with 5% CO_2_).

The BoHV-1/BarthaNu/67 strain (HVRIIBRV0004) was provided by China Veterinary Culture Collection Center (CVCC) [6]. Viral titers were determined using the Reed–Muench method and expressed as the 50% tissue culture infectious dose per mL (TCID_50_ / mL). MDBK cells were infected with BoHV-1 at 0.1 MOI, and cultured for 4, 8, 12, and 24 h [37].

BoHV-1 was inactivated by exposing the virus stock to 254-nm short-wave UV radiation in a 100-mm culture plate on ice for 12 h. The cells were harvested and preserved at −80 °C.

### 2.2. Antibodies and Reagents

Antibodies against GRP78 (ab21685), ATF6 (ab122897), p-PERK (ab192591) and p-eIF2α (Ser51) (ab32157) were obtained from Abcam (Cambridge, UK). Antibodies against total PERK (sc-13073) were purchased from Santa Cruz Biotechnology (Dallas, TX, USA). Antibodies against p-IRE1 (S724) (CY5605), eIF2α (AB3335), and β-actin (AB0061) were purchased from Abways Technology (Shanghai, China). Antibodies against total IRE1 (3294S) were purchased from Cell Signaling Technology (Danvers, MA, USA). Thapsigargin (Tg) (Abcam, ab120286), GSK2606414 (HY-18072) (MedChem Express, Monmouth Junction, NJ, USA), APY29 (HY-17537) (MedChem Express, Monmouth Junction, NJ, USA) and 4μ8C (HY-19707) (MedChem Express, Monmouth Junction, NJ, USA) were dissolved in DMSO.

### 2.3. RNA Interference

Small interfering RNAs (siRNAs) against ATF6, PERK, and IRE1 were designed to target three different coding regions of each given gene, and were purchased from RiboBio (Guangzhou, China). MDBK cells were seeded at 80% confluence in a 6-well plate (Corning Inc., Corning, NY, USA). The si-ATF6, si-PERK, si-IRE1, and si-Control were transfected using Attractene Transfection Reagent (Qiagen, Hilden, Germany) according to the manufacturer’s instructions. The cells were infected with BoHV-1 after 48 h post-transfection, followed by harvesting the sample for protein and virus titration analysis. The sequence of each siRNA is listed in Table 1.

### 2.4. Cell Viability Measurement

Cell viability was determined by using the cell counting Kit-8 (Boster, Wuhan, China) according to the manufacturer’s protocols [38].

### 2.5. XBP1 mRNA Splicing Assay

Total cellular RNA was purified using Trizol reagent (Thermo Fisher Scientific, Hampton, NH, USA) according to the manufacturer’s instructions. RT-PCR was performed by using PrimeScript™ One Step RT-PCR Kit Ver.2 (TaKaRa Bio Inc., Otsu, Japan) [39,40,41]. The XBP1 gene was amplified by PCR with XBP1-F: 5′-CTGAAAAACAGAGTAGCAGCTCAGA-3′ and XBP1-R: 5′-TCAGTTCATTAATGGCTTCCAGC-3′. The spliced (XBP1s) and unsliced (XBP1u) forms were analyzed by digesting the RT-PCR products with the restriction enzyme Pst I (FD0615) (Thermo Fisher Scientific, Hampton, NH, USA). Followed by purification of digested PCR products with 1.5% agarose gel electrophoresis and analysis by using the gel imaging system (Federal bioproducts Inc., Manufacturer, UK) [28,42,43].

### 2.6. Quantitative-PCR (qPCR) Analysis

To detect the UPR induced by BoHV-1 infection, MDBK cells were challenged with BoHV-1 at an MOI of 0.1, and the cells were collected at 0, 4, 8, 12, and 24 h post-infection (hpi). To detect the efficiency of siRNA-mediated gene silencing, MDBK cells were transfected with 50 nM of siRNA using Attractene Transfection Reagent (Qiagen, Hilden, Germany) and were collected at 24 h post transfection. Total cellular RNA was reverse transcribed to make cDNA by PrimeScript™ RT Master Mix (Perfect Real Time) (TaKaRa Bio Inc., Otsu, Japan) and PrimeScript RT reagent (TaKaRa Bio Inc., Otsu, Japan) [44,45,46]. Relative fold induction was calculated with the 2^−ΔΔCt^ method. The primers for qPCR analysis of UPR gene expression are listed in Table 2.

### 2.7. Western Blot

MDBK cells were infected with BoHV-1 and harvested at the indicated time points. The cells were lysed with the cell lysis buffer (50 mM Tris-HCl, pH 7.4, 150 mM NaCl, 1% Triton X-100, 1% sodium deoxycholate, 2 mM EDTA, 0.1% SDS, 5 mM sodium orthovanadate) supplemented with 0.1 mM phenylmethylsulfonyl fluoride (PMSF) and the protease inhibitor cocktail (MedChem Express, Princeton, NJ, USA). Briefly, about 20 to 30 µg of the cell lysates was used to separate proteins on 10% SDS-PAGE, and the proteins isolated on the gel were transferred to PVDF membranes, which were then blocked with 5% nonfat dry milk in Tris-Buffered Saline Tween-20 (TBST, 20 mM Tris-HCl, pH 7.4, 150 mM NaCl, 0.1% Tween 20). The membranes were incubated with appropriate primary antibodies diluted in TBST supplemented with 5% nonfat dry milk overnight at 4 °C, and then washed five times with TBST. Finally, the incubated membranes were treated with a horseradish peroxidase-conjugated secondary antibody for 2 h as previously described [48,49,50]. Protein bands were quantified and analyzed by densitometry using AlphaView software (version 3.4; ProteinSimple, Santa Clara, CA, USA).

### 2.8. Virus Replication Assay

Virus replication assay was performed as previously described [51]. To test whether inhibition of PERK and IRE1 affect BoHV-1 binding to MDBK cells, the cells in a 6-well plate were treated with different inhibitors for 1 h at 37 °C to block PERK and IRE1 pathways respectively. Next, MDBK cells were infected with BoHV-1 (0.1 MOI) for 1 h at 4 °C to allow the viruses to adsorb to the cell membrane. Subsequently, the cells were washed three times with ice-cold PBS and underwent two freeze–thaw processes. The virus titer was determined on MDBK cells.

To identify the effects of PERK and IRE1 inhibition on the BoHV-1 entry process, confluent MDBK cells in a 6-well plate were infected with BoHV-1 (0.1 MOI) at 4 °C for 1 h. The infected cells were washed three times with ice-cold PBS and then treated with the inhibitors for 1 h at 37 °C to allow the viruses to enter the cells. The MDBK cells were then washed three times with ice-cold PBS and treated with citrate buffer (135 mM NaCl, 40 mM citric acid, 10 mM KCl, pH 3.0) for 1 min to inactivate unpenetrated virions [52]. Finally, the fresh DMEM medium with 2% FBS was replaced and continuously incubated for 24 h at 37 °C. In parallel, to test whether PERK and IRE1 inhibition affect the post-entry stage of the BoHV-1 life cycle, MDBK cells were exposed to BoHV-1 (0.1 MOI) for 1 h at 4 °C and then moved to 37 °C for 1 h to allow the virus to enter the cells. Subsequently, the cells were treated with citrate buffer for 1 min, then incubated with fresh DMEM supplemented with 2% FBS and different inhibitors for 24 h at 37 °C [53]. The virus titer was determined on MDBK cells.

### 2.9. Statistical Analyses

All the graphs and statistical analyses were created by GraphPad Prism software (version 8.0; San Diego, CA, USA). The results are presented as the mean value ± standard deviations (SD) from at least three independent experiments, and a *p* value below 0.05 was considered statistically significant (* *p* < 0.05; ** *p* < 0.01).

## 3. Results

### 3.1. BoHV-1 Infection Triggers ER Stress in MDBK Cells

**Figure 1:**
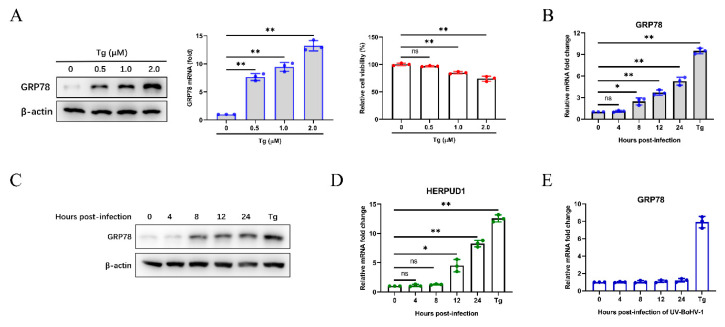
BoHV-1 infection triggers ER stress in MDBK cells. (**A**) The expression of GRP78 in MDBK cells treated with different concentrations of ER stress inducer Tg for 24 h was detected by western blotting and qPCR, and the cell counting kit 8 (CCK-8) assays were performed to analyze the viability of the MDBK cells. (**B**) A time-dependent increase of GRP78 mRNA levels was revealed by qPCR in MDBK cells infected with BoHV-1 at an MOI of 0.1 at different time points (0 to 24 h) after infection. Tg (0.5 μM) was used as a positive control for ER stress. (**C**) The GRP78 protein levels were measured by western blotting at 0 to 24 hpi, and Tg (0.5 μM) was used as the positive control. (**D**) qPCR analysis of HERPUD1 mRNA levels in MDBK cells at 0 to 24 h after BoHV-1 infection. (**E**) qPCR analysis of GRP78 mRNA levels in MDBK cells infected with UV-inactivated BoHV-1 or treated with Tg (0.5 μM). The graph shows the levels of GRP78 or HERPUD1 normalized against β-actin (**A**,**B**,**D**,**E**). The mean ± SD of data from three independent experiments are shown; * *p* < 0.05, ** *p* < 0.01.

We initially analyzed the expression of GRP78 induced by different concentrations of the ER stress inducer thapsigargin (Tg), and its effect on cell viability (Figure 1A). To investigate whether BoHV-1 infection induces cellular ER stress, we monitored the expression of GRP78 in MDBK cells, and Tg (0.5 µM) was used as a positive control. The results showed that GRP78 mRNA levels were upregulated from 8 to 24 h post-infection (hpi) (Figure 1B). The upregulated GRP78 expression was confirmed by western blotting compared with cells infected at 0 h, indicating the UPR activation in BoHV-1 infected MDBK cells (Figure 1C). We further confirmed the results by analyzing the mRNA expression of HERPUD1, which is an ER stress-responsive marker [54] (Figure 1D). Furthermore, UV-inactivated BoHV-1 did not trigger the upregulation of GRP78 mRNA levels, and the results further verified that BoHV-1 infection rather than viral proteins, could induce ER stress in MDBK cells (Figure 1E).

### 3.2. Bohv-1 Induces UPR Through All Three Signaling Branches

**Figure 2:**
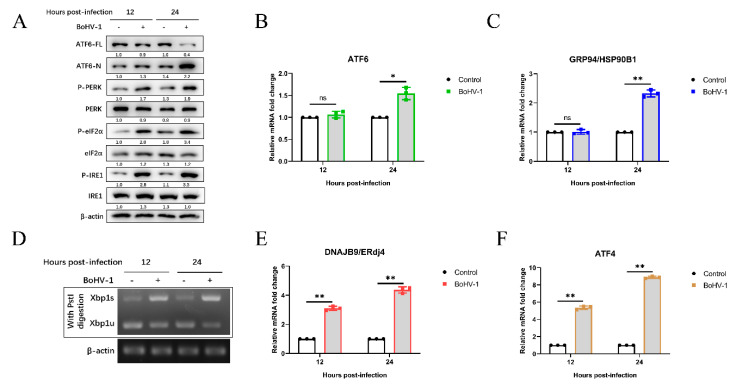
BoHV-1 induces UPR through all three signaling branches. (**A**) Immunoblot analysis of ATF6-FL, ATF6-N, total PERK, eIF2α, and IRE1, phosphorylated IRE1, PERK, and eIF2α protein levels in MDBK cells after 12 and 24 h of BoHV-1 infection at an MOI of 0.1. (**B**,**C**,**E**,**F**) qPCR analysis of ATF6, GRP94, ERdj4, and ATF4 mRNA expression in MDBK cells after 12 and 24 h of BoHV-1 infection (0.1 MOI). The graph shows the levels of the genes normalized against β-actin and the data are shown as fold changes and were normalized to control cell data. (**D**) XBP1 splicing assay. First, MDBK cells were infected with BoHV-1 at 0.1 MOI at 12 or 24 hpi and harvested for RT-PCR analysis of XBP1 mRNA splicing. The production of XBP1 was digested with the restriction enzyme Pst I, and then the digested PCR products were separated by 1.5% agarose gel electrophoresis. The mean ± SD of data from three independent experiments are shown; * *p* < 0.05, ** *p* < 0.01.

Next, we determined which UPR signaling pathways (PERK, IRE1, and ATF6) were activated by BoHV-1 infection. The expressions of UPR-related genes were examined using qPCR and western blotting at the indicated time points. During ER stress, the 90-kDa ATF6 (ATF6-FL) is cleaved into the active form of 50-kDa (ATF6-N). We observed that the ATF6-N was significantly increased at 24 hpi (Figure 2A). Activation of the ATF6 pathway can induce the up-regulation of GRP94/HSP90B1 mRNA levels [55,56]. The ATF6 and GRP94 mRNA levels were increased approximately 2-fold at 24 hpi. These results indicated that the ATF6 pathway was activated during BoHV-1 infection.

To investigate the effect of BoHV-1 infection on the IRE1-XBP1 pathway, we measured the protein levels of phosphorylated IRE1, total IRE1, and the mRNA levels of spliced XBP1 (XBP1s) and unspliced XBP1 (XBP1u). The results showed that compared with mock infection, BoHV-1 infection significantly upregulated the phosphorylated IRE1 levels at 12 and 24 hpi (Figure 2A). IRE1 activation cleaved a 26-base intron segment of the XBP1 mRNA to XBP1s, which activated the transcription of genes to improve cellular protein-folding capacity [57]. The XBP1 cDNA was amplified by reverse transcriptase (RT)-PCR and followed by enzyme digestion with Pst I. The recognition site of Pst I is located in the 26-base intron segment of XBP1 cDNA, which was removed by IRE1-mediated splicing [28,43,57]. We found that XBP1s levels were upregulated after BoHV-1 infection at 12 to 24 hpi, and the levels of XBP1u were decreased (Figure 2D). Meanwhile, compared with mock infection, BoHV-1 infection induced higher mRNA levels of the XBP1s downstream target ER-localized DnaJ homologue 4 (ERdj4) gene [58], which further confirmed the XBP1 splicing (Figure 2E). These results showed that the BoHV-1 infection induced the IRE1-XBP1 pathway.

Both phosphorylation of PERK and eIF2α were increased in BoHV-1-infected MDBK cells compared with control samples at 12 to 24 hpi (Figure 2A). Moreover, we found that the mRNA levels of ATF4 were upregulated at 12 and 24 hpi during BoHV-1 infection (Figure 2F), indicating the activation of the PERK pathway. Altogether, these data demonstrated that BoHV-1 infection activates UPR through all three signaling branches.

### 3.3. The Virus-Induced ATF6 Pathway Does Not Affect Bohv-1 Replication

**Figure 3:**
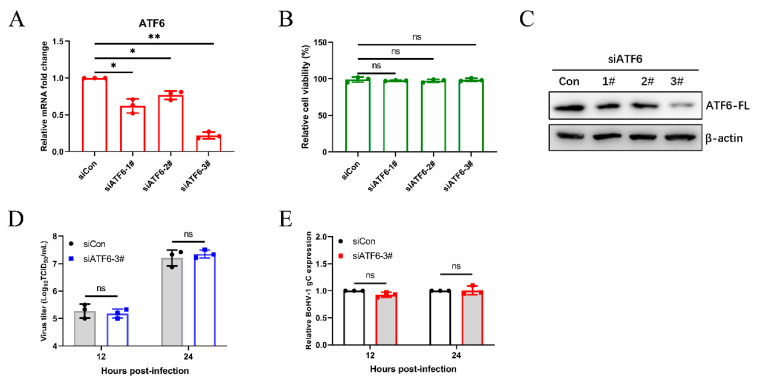
The virus-induced ATF6 pathway does not affect BoHV1 replication. MDBK cells were transfected with siRNA targeting ATF6 or scrambled siRNA (siCon). (**A**) The silencing efficiency of ATF6 was measured by qPCR in MDBK cells at 24 h post-transfection. (**B**) The CCK-8 assays were used to analyze the viability of MDBK cells 24 h after transfection. (**C**) Immunoblot analysis of the silencing efficiency of ATF6 at 48 h post-transfection. (**D**,**E**) At 48 h siATF6-3# post-transfection, MDBK cells were infected with BoHV-1 (0.1 MOI) and harvested at the indicated time points for virus titration and the qPCR analyses. The mean ± SD of data from three independent experiments are shown; * *p* < 0.05, ** *p* < 0.01.

We next analyzed the effects of different UPR pathways on BoHV-1 replication. We examined BoHV-1 infection in the ATF6 knockdown MDBK cells by using siRNAs duplexes and by transfecting cells. After transfecting siRNA into MDBK cells for 24 h, the silencing efficiency of siRNAs on ATF6 mRNA levels was determined by qPCR (Figure 3A), and it was determined that transfection of siRNA into MDBK cells did not effect cell viability (Figure 3B). Subsequently, MDBK cells were transfected with siRNA of ATF6 for 48 h, and then the efficiency of ATF6 knockdown by different siRNAs was confirmed using western blotting. We confirmed that ATF6-3# has the best silencing effect by analyzing the protein levels of ATF6-FL (Figure 3C). Then, we measured the virus titer and the BoHV-1 glycoprotein C (gC) transcript showed that silencing ATF6 did not affect BoHV-1 replication in MDBK cells (Figure 3D,E). These data demonstrated that the ATF6 signaling pathway does not affect BoHV-1 proliferation in MDBK cells.

### 3.4. The BoHV1-Activated PERK Pathway Facilitates Viral Proliferation

**Figure 4:**
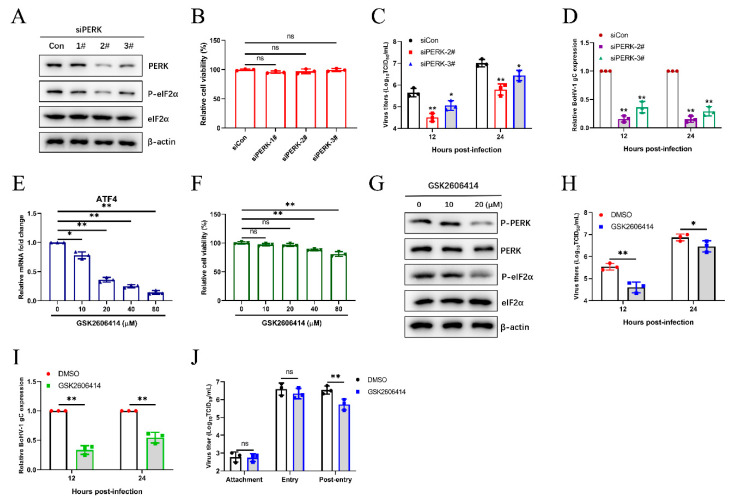
The BoHV1-activated PERK pathway facilitates viral proliferation. (**A**) MDBK cells were transfected with siRNAs targeting PERK for 48 h, and then cells were infected with BoHV-1. Immunoblot analysis of the silencing efficiency of PERK at 24 hpi. (**B**) The CCK-8 assays were performed to analyze the MDBK cell viability. (**C**,**D**) At 48 h post siRNA transfection, MDBK cells were infected with BoHV-1 (0.1 MOI). The viral titers of BoHV-1 and the relative expression of BoHV-1 gC were detected at the indicated time points. (**E**) The efficiency of GSK2606414 in inhibiting the PERK pathway was evaluated by detecting the ATF4 mRNA levels in MDBK cells. (**F**) The cytotoxicity of GSK2606414 was measured using a CCK-8 assay. (**G**) Immunoblot analysis of total PERK, total eIF2α, phosphorylated PERK, and phosphorylated eIF2α in MDBK cells treated with different concentrations of GSK2606414. (**H**,**I**) The viral titers and gene expression of BoHV-1 in MDBK cells treated with GSK2606414 (20 µM) or DMSO at 12 and 24 hpi were determined. (**J**) Effect of PERK inhibition on BoHV-1 replication stages. MDBK cells infected with BoHV-1 (0.1 MOI) were treated with GSK2606414 (20 μM) at the virus binding, virus entry, or post-entry process, respectively. The viral titers were determined in MDBK cells. The mean ± SD of data from three independent experiments are shown; * *p* < 0.05, ** *p* < 0.01.

To further investigate the roles of the PERK pathway on BoHV-1 replication, we initially selected the appropriate siRNAs for silencing PERK in MDBK cells by western blotting and CCK-8 assay (Figure 4A,B). Subsequently, we further determined the viral titration and gene expression in the MDBK virus-infected PERK inhibition cells, which were significantly lower than that in control cells at 12 and 24 hpi (Figure 4C,D). We then confirmed the impact of PERK on viral replication by using the PERK-specific inhibitor GSK2606414 to disrupt the PERK signaling pathway. Previous research revealed that ATF4 mRNA transcript levels could be elevated in response to the PERK pathway [59,60,61]. The PERK inhibition by GSK2606414 was detected by qPCR to analyze ATF4 mRNA levels (Figure 4E). The effects of GSK2606414 on the viability of MDBK cells were determined (Figure 4F). PERK inhibition by GSK2606414 was confirmed by western blotting *p*-PERK, PERK, *p*-eIF2α, and eIF2α (Figure 4G). Consistent with the results of siRNA treatment, PERK inhibition (GSK2606414 20 µM) inhibited viral proliferation during BoHV1 infection (Figure 4H,I). Next, we identified which stage(s) of the BoHV-1 replication, including virus binding, entry, and post-entry stages, was affected by the PERK pathway. The results showed that inhibiting the PERK pathway affects the post-entry stage of viral replication in MDBK cells (Figure 4J). These collective data suggest that the PERK pathway could promote BoHV-1 proliferation via affecting the virus post-entry process in MDBK cells.

### 3.5. The IRE1 Pathway Induced by Bohv-1 Infection Promotes Viral Replication in MDBK Cells

**Figure 5:**
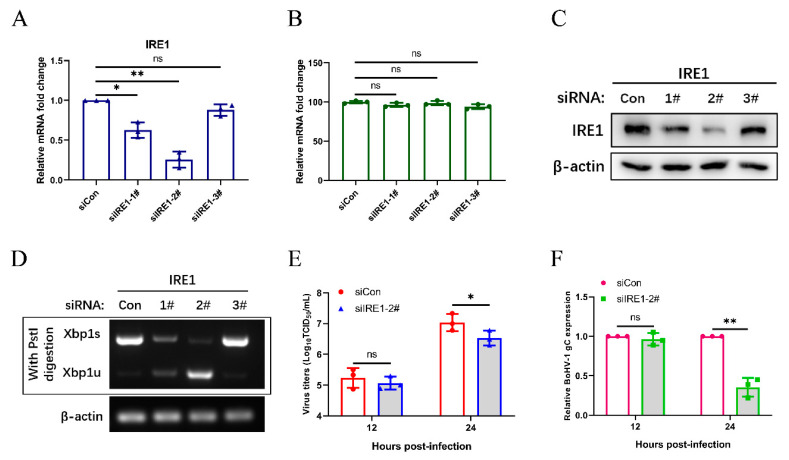
The IRE1 pathway induced by BoHV-1 infection promotes viral replication in MDBK cells. (**A**) The silencing efficiency of IRE1 was measured by qPCR in MDBK cells at 24 h post-transfection. (**B**) The CCK-8 assays were used to analyze the viability of MDBK cells 24 h after transfection. (**C**) Immunoblot analysis of the silencing efficiency of IRE1 at 48 h post-transfection. (**D**) At 48 h siRNA duplexes post transfection, MDBK cells were challenged with BoHV-1 (0.1 MOI) and then harvested at 24 hpi for XBP1 splicing assay. (**E**,**F**) At 48 h siCon or siIRE1 post transfection, MDBK cells were infected with BoHV-1 (0.1 MOI) and harvested at the indicated time points for virus titration analyses and viral gene expression analyses. The mean ± SD of data from three independent experiments are shown; * *p* < 0.05, ** *p* < 0.01.

We further analyzed the effects of the IRE1 pathway on BoHV-1 replication. The knockdown efficiency of IRE1 siRNAs was determined by qPCR (Figure 5A), and the CCK-8 assay was put to use for measuring the cell viability in response to transfection with IRE1 siRNA (Figure 5B). The knockdown efficiency of siRNA targeting IRE1 was confirmed by western blotting and XBP1 splicing (Figure 5C,D). The siIRE1-2# was selected for subsequent experiments. After 48 h post transfection, MDBK cells were infected with BoHV-1 with an MOI of 0.1, harvested at 12 and 24 hpi, and viral replication was assessed by measuring viral titers and mRNA expression levels of the gC gene (Figure 5E,F). The results showed that the silencing of IRE1 inhibits virus replication at 24 hpi, suggests that the IRE1 pathway may promote BoHV-1 replication.

### 3.6. Bohv-1 Replication is Enhanced by Inhibiting IRE1 Rnase Activity in MDBK Cells

**Figure 6:**
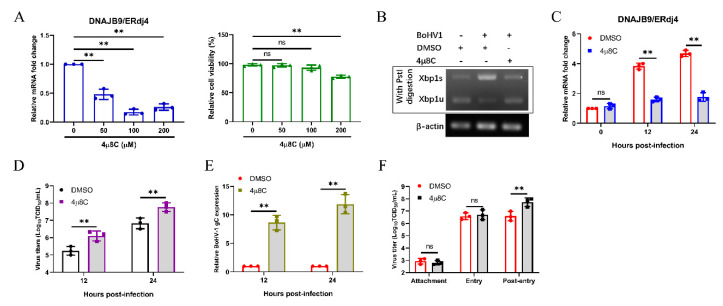
BoHV-1 replication is enhanced by inhibiting IRE1 RNase activity in MDBK cells. (**A**) The efficiency of 4μ8C in inhibiting the IRE1 RNase activity was evaluated by detecting the ERdj4 mRNA levels in MDBK cells. The cytotoxicity of 4μ8C was measured using a CCK-8 assay. (**B**–**E**) MDBK cells were treated with 4μ8C (100 µM) or DMSO, followed by infection with BoHV-1 (0.1 MOI). (**B**) MDBK cells were harvested at 24 hpi and subjected to XBP1 splicing assay. (**C**) MDBK cells were harvested at 0, 12, and 24 hpi, and qPCR analysis of ERdj4 mRNA levels. (**D**) MDBK cells were harvested at 12 and 24 hpi and viral titers of BoHV-1 were determined. (**E**) qPCR analysis of the BoHV-1 gC expression in MDBK cells at 12 and 24 hpi. (**F**) Effect of IRE1 RNase activity inhibition on BoHV-1 replication stages. The mean ± SD of data from three independent experiments are shown; * *p* < 0.05, ** *p* < 0.01.

IRE1 is a unique enzyme, possessing both RNase and kinase activity [62]. As reported, 4μ8C, an IRE1-specific inhibitor, inhibits IRE1 splicing of XBP1 mRNA to reduce subsequent ERdj4 expression, but does not block IRE1 autophosphorylation [63,64,65]. To investigate the effect of the IRE1 RNase activity on BoHV-1 replication, we used 4μ8C to inhibit the IRE1 RNase activity. First, we confirmed the mRNA expression of DNAJB9/ERdj4 by qPCR to confirm the inhibitory effect of 4μ8C, and the standard CCK-8 assay was applied to investigate the effect of 4μ8C on cell viability (Figure 6A). The inhibitory effect of 4μ8C (100 μM) was also detected by XBP1 splicing assay (Figure 6B). Next, we measured the expression of DNAJB9/ERdj4 by qPCR to further confirm the inhibitory effect of 4μ8C on IRE1 within 24 h of BoHV1 infection (Figure 6C). Interestingly, the results showed that inhibiting the IRE1 RNase activity facilitates BoHV-1 replication (Figure 6D). The results of the BoHV-1 gC transcript by qPCR also showed that IRE1 RNase activity inhibition supports the proliferation of BoHV-1 (Figure 6E). Further studies found that IRE1 RNase activity inhibition affected the post-entry stage of BoHV-1 replication (Figure 6F). These results demonstrated that inhibiting IRE1 RNase activity could promote virus replication at the post-entry step of the BoHV-1 life cycle.

### 3.7. Inhibition of IRE1 Kinase Activity and Promotion of Rnase Activity Decrease Bohv-1 Replication

**Figure 7:**
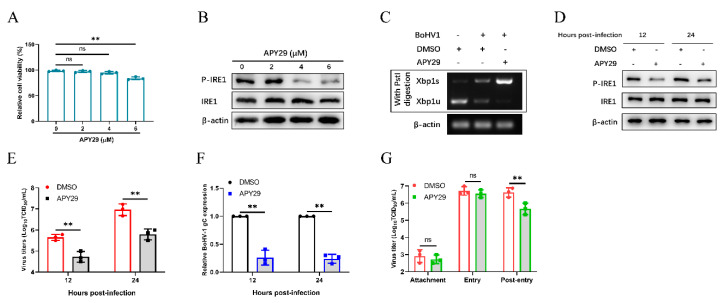
Inhibition of IRE1 kinase activity and promotion of RNase activity decrease BoHV-1 replication. (**A**) The cytotoxicity of APY29 was measured using a CCK-8 assay. (**B**) MDBK cells were treated with different concentrations of APY29, and then challenged with BoHV-1. Immunoblot analysis of total IRE1 and phosphorylated IRE1 in MDBK cells at 24 hpi. (**C**) MDBK cells were treated with APY29 (4 µM) or DMSO, and followed by infection with BoHV-1. Cells were harvested at 24 hpi and subjected to XBP1 splicing assay. (**D**–**F**) MDBK cells treated with APY29 (4 μM) or DMSO were challenged with BoHV-1 (0.1 MOI). Cells were harvested at 12 and 24 hpi. (**D**) Immunoblot analysis of the IRE1 kinase activity at the indicated time points. (**E**) The viral titers of BoHV-1 in MDBK cells at 12 and 24 hpi were determined. (**F**) qPCR analysis of the BoHV-1 gC expression in MDBK cells at the indicated time points. (**G**) Effect of IRE1 kinase activity inhibition and RNase activity enhancement on BoHV-1 replication stages. The mean ± SD of data from three independent experiments are shown; * *p* < 0.05, ** *p* < 0.01.

The phosphorylation level of IRE1 is generally accepted as the reliable indicator of IRE1 kinase activity [63,66,67]. APY29, a class I IRE1 inhibitor, could bind to the ATP binding site of IRE1 to inhibit its autophosphorylation and enhance its RNase activity [66,68]. Therefore, we evaluated the effect of IRE1 kinase activity inhibition and RNase activity enhancement on BoHV-1 replication by treating MDBK cells with an inhibitor (APY29). Firstly, the effects of different APY29 concentrations on MDBK cell viability were examined by the CCK-8 kit, the results showed that the optimum concentration was 4 µM (Figure 7A). Secondly, it was shown that APY29 (4 µM) could inhibit the phosphorylation of IRE1 (Figure 7B) and enhance the activity of the IRE1 RNase in MDBK cells infected with BoHV-1 at 24 hpi (Figure 7C). We next confirmed the inhibitory effect of the APY29 (4 µM) on IRE1 kinase activity during BoHV-1 infection by Western blot analysis (Figure 7D). In addition, we found that treating cells with APY29 significantly reduced BoHV-1 replication at 12 and 24 hpi (Figure 7E,F), which mainly occurred in the post-entry stage of viral replication (Figure 7G). These data suggested that inhibition of IRE1 kinase activity and enhancement of RNase activity could inhibit the replication of BoHV-1 in the virus post-entry phase.

## 4. Discussion

It has been widely reported that, during viral replication, large amounts of unfolded proteins accumulate in the ER lumen, which usually causes ER stress and UPR activation to maintain protein homeostasis and cell survival. Many reports have focused on the induction mechanisms of UPR activation and the effect of UPR on the viral replication [69]. ZIKV infection triggers UPR activation with IRE1 and PERK branches, and the UPR activation influences viral replication [70]. The E3-19K glycoprotein of human adenovirus (AdV) specifically induces the IRE1-XBP1 pathway, which enhances AdV infection, and XBP1s could bind to the E1A-enhancer/promoter to improve E1A transcription and lytic infection [71]. However, previous studies have not shown whether BoHV-1 infection induces ER stress and UPR. Therefore, we measured the expression of the ER stress marker protein GRP78 in MDBK cells during the BoHV-1 infection. We first confirmed the GRP78 expression induced by ER stress inducer Tg in MDBK cells, and determined the optimum concentration (Figure 1A). Our previous research found that after BoHV-1 infection of MDBK cells with 0.1 multiplicity of infection (MOI) for 24 h, there will be an obvious cytopathic effect (CPE) and the virus titer will reach its peak [37,72]. Next, we found that both mRNA and protein levels of GRP78 significantly increased compared with uninfected control cells (Figure 1B,C). Moreover, the HERPUD1 mRNA levels were up-regulated during BoHV-1 infection (Figure 1D), and inactivated BoHV-1 could not cause the expression of GRP78 changes (Figure 1E). These results indicate that BoHV-1 infection triggers ER stress in MDBK cells.

More recently, it has been reported that herpesvirus family members trigger UPR to affect viral replication. Examination of the UPR pathway during pseudorabies virus (PRV) infection revealed that IRE1-XBP1 and eIF2α-ATF4 were activated, and overexpression of GRP78 could promote PRV production [73]. KSHV lytic replication activates all three UPR sensors, and KSHV requires UPR sensor protein activation to replicate [74]. Here, we found that BoHV-1 infection could also induce three pathways of UPR in MDBK cells. BoHV-1 infection upregulated the cleaved ATF6 level at 24 hpi (Figure 2A). Compared with uninfected cells, the mRNA levels of ATF6 and GRP94 were significantly increased at 24 hpi, suggesting that the ATF6 pathway was activated at 24 hpi (Figure 2B,C). Moreover, we found that the phosphorylation level of IRE1 was upregulated at 12 and 24 hpi (Figure 2A). In addition, XBP1s and DNAJB9/ERdj4 mRNA levels in BoHV-1-infected MDBK cells were significantly higher than in control cells (Figure 2D,E), indicating that BoHV-1 infection induced the IRE1 pathway. Furthermore, the phosphorylation level of PERK and eIF2α were upregulated (Figure 2A), the results of the qPCR analysis of ATF4 also suggested that the PERK pathway was activated during BoHV-1 infection (Figure 2F). These findings are the same as the results in Marek’s disease virus (MDV), which is a member of the herpesvirus family and activates UPR pathways in cell culture [56]. Collectively, our data suggest that BoHV-1 triggers UPR through all three signaling branches.

Many replication strategies have been evolved by viruses that are facilitated by using UPR. For example, HSV-1 regulates the IRE1 pathway to inhibit XBP1s activation [33], and the induction of PERK-eIF2α-DDIT3 promotes apoptosis and cytokine secretion to support NDV proliferation [69]. The specific roles of the three UPR branches in viral replication are not the same. In this study, we found that the ATF6 pathway could not modulate BoHV-1 proliferation according to the results of the siRNA knockdown (Figure 3). The PERK branch of UPR is closely related to viral replication [75,76,77]. It has been reported that PERK activation induced by foot-and-mouth disease virus (FMDV) suppresses the antiviral interferon response to promote viral replication [78]. Here, we demonstrated that knockdown and chemical inhibition of PERK suppressed BoHV-1 replication via affecting the virus post-entry (Figure 4). However, PERK-eIF2α-mediated reduction in global translation is thought to be an antiviral response that restricts replication [43,79]. To survive, some viruses have evolved specific mechanisms to regulate the PERK pathway. HCMV activation of the PERK pathway could promote viral proliferation by activating SREBP1 to regulate intramembrane proteolysis [80]. We thus speculate that the PERK pathway induced by BoHV-1 infection may affect virus replication by regulating autophagy, innate immunity, or lipid synthesis at the post-entry step of the BoHV-1 life cycle. Next, we need future studies to examine the specific mechanism of the PERK pathway of UPR in modulating the BoHV-1 replication.

Previous studies have shown that the IRE1-XBP1 pathway induced by influenza A virus or classical swine fever virus could facilitate viral replication [81,82]. The IRE1-XBP1 pathway could promote virus replication by enhancing the folding ability of ER protein and membrane biosynthesis [83]. In this study, we found that the IRE1 pathway induced by BoHV-1 infection also promotes viral replication in MDBK cells (Figure 5). We further used specific inhibitors to block the kinase or RNase activity of IRE1 to examine the effect of its kinase or RNase activity inhibition on BoHV-1 replication. Using APY29 to inhibit IRE1 kinase activity and enhance RNase activity could inhibit BoHV-1 replication at the stage of virus post-entry (Figure 7). Interestingly, we found that IRE1 could promote BoHV-1 replication (Figure 5). However, inhibiting the IRE1 RNase activity promoted BoHV-1 replication, indicating that the RNase activity of IRE1 could inhibit BoHV-1 replication (Figure 6). Taken together, we speculated that the IRE1 kinase activity could promote BoHV-1 replication, and play a more important role than the RNase activity in ultimately enabling IRE1 to promote BoHV-1 replication. It has been reported that IRE1 kinase activity promotes HSV-1 replication by activating the JNK pathway, and IRE1 RNase activity could inhibit viral replication [33]. IRE1 RNase activity promotes ER redistribution to contribute to ZIKV replication [84]. However, we found that IRE1 RNase activity inhibits BoHV-1 infection. It may be a balance mechanism evolved by cells to limit the effect of IRE1 kinase activity on BoHV-1 replication.

In conclusion, we reported that BoHV-1 infection induces ER stress to activate all three branches of the UPR in MDBK cells. The data showed that the ATF6 pathway does not affect BoHV-1 replication in MDBK cells, and PERK and IRE1 pathways were beneficial to viral replication. Our results further demonstrated that the RNase activity inhibition of IRE1 promoted viral replication and the IRE1 kinase activity inhibition and RNase activity promotion were detrimental to BoHV-1 replication at the post-entry step of the BoHV-1 life cycle. These results provide new insights to understand the molecular mechanism of BoHV-1 pathogenesis.

## Figures and Tables

**Table 1:** Small interfering RNA (siRNA) sequence.

Name	Sequences (5′ to 3′)
siATF6-1#	ACAGAAACCACTAGTATCA
siATF6-2#	CTCATCAACTCAGCATGTT
siATF6-3#	CAAGCCTTTATTACTTCCA
siPERK-1#	GATCCTAACTGATGTAAGA
siPERK-2#	GGTTGATGACTGCAATTAT
siPERK-3#	GCTGTATCTGCAATCATCA
siIRE1-1#	GCTTTGAGGAGGTCATTGA
siIRE1-2#	CTTCTACTACGTGATATCT
siIRE1-3#	GGAAATTCAGAACCTATAA

**Table 2:** The primers used for quantitative-PCR (qPCR) analysis.

Name	Sequences (5′ to 3′)
GRP78-F	CGGAGGAGGAGGACAAGAAGGAG C
GRP78-R	ATAAGACGGCGTGATGCGGTTG
HERPUD1-F	ATCAGAACGCTGCTCCACAAGTG
HERPUD1-R	TAGCGGCTGAGTAGGTCCAATCC
ATF6-F	GAGGAGCAAGACACATCGGATGAC
ATF6-R	TGACAGGGAGGCGGAGGAATATAG
GRP94/HSP90B1-F	CAAGATCGAGAAGGCTGTGGTGTC
GRP94/HSP90B1-F	GATGTCCTTGCCTGTCTGGTATGC
ATF4-F	CCCAAACCCTACGACCCTCCTG
ATF4-R	TCCTGTTCCGCCCTCTTCTTCTG
DNAJB9/ERdj4-F	GGAGCGCCAAGTCAAGAAGG
DNAJB9/ERdj4-R	GCTTCAGCATCAGGGCTCTT
BoHV-1 gC-F	ATGTTAGCGCTCTGGAACC
BoHV-1 gC-R	CTTTACGGTCGACGACTCC [47]
β-actin-F	CCATCGGCAATGAGCGGTTCC
β-actin-R	CGTGTTGGCGTAGAGGTCCTTG
